# Prevalence of Transfusion-transmitted Virus (TTV) Infection and its Association with Renal Post-transplantation Complications in Iran

**Published:** 2018-08-01

**Authors:** H. Akbari, A. Piroozmand, E. Dadgostar, H. Nikoueinejad, Z. Chitsazian, B. Einollahi, J. Amini Mahabadi

**Affiliations:** 1 Department of Biostatistics and Epidemiology, Kashan University of Medical Sciences, Kashan, Iran; 2 Department of Microbiology, Faculty of Medicine, Kashan University of Medical Sciences, Kashan, Iran; 3 Autoimmune Diseases Research Center, Kashan University of Medical Sciences, Kashan, Iran; 4 Halal Research Center of IRI, FDA, Tehran, Iran; 5 Nephrology and Urology Research Center, Baqiyatallah University of Medical Sciences, Tehran, Iran; 6 Department of Internal Medicine, Kashan University of Medical Sciences, Kashan, Iran; 7 Gametogenesis Research center, Kashan University of Medical Sciences, Kashan, Iran

## Abstract

Background:

Transfusion-transmitted virus (TTV) is a single-stranded DNA virus. Renal transplant patients have a higher risk of TTV infection.

Objective:

To evaluate the prevalence of TTV and its correlation with post-renal transplantation complications in a population of Iranian patients.

Methods:

A cross-sectional study was performed on 120 renal transplant recipients. TTV infection in the peripheral blood samples was detected by semi-nested polymerase chain reaction (semi-nested PCR). Then, the relationship between TTV and renal post-transplant complications was examined.

Results:

34.2% renal transplant recipients were positive for TTV. There was a significant correlation between the presence of TTV and diabetes, acute transplant rejection, and urinary tract infection. We did not find any direct correlation between the presence of TTV infection and hypertension, hyperlipidemia, respiratory tract infection, and cytomegalovirus infection.

Conclusion:

We found an increased rate of TTV infection in renal transplant recipients associated with post-transplantation complications. TTV may be an important risk factor for some post-renal transplantation complications.

## INTRODUCTION

Kidney transplantation, the most effective treatment for chronic renal failure [1], is strongly increasing all over the world [2]. Aside from the side effects, which the transplantation imposes on patients, the side effects of weakened immune system, to prevent transplant rejection, faces the patients with problems such as different bacterial, fungal and viral infections.

Transfusion-transmitted virus (TTV, or torque teno virus) is a nude circular single-stranded DNA virus discovered by Nishizawa, *et al*, in Japanese patients’ serum with post-transfusion hepatitis of unknown origin, in 1997 [3]. TTV is prevalent worldwide prevalence. Different studies using sensitive PCR systems indicate high prevalence of TTV [3, 4].

TTV accompanies with some clinical conditions. For example, TTV prevalence is 30%–42.9% in hemodialysis patients [5, 6], 20% in intravenous drug abusers [7], 75% in hemophiliacs [8], 46.7% in patients with hepatocellular carcinoma, 40% in cirrhotic patients (compared to 36.7% in healthy people) [9], 46% in non-AG viral liver infections (compared to 12% in healthy people), 48% in patients with fulminant hepatitis [10], and 84.2% in HIV-infected (compared to 63% of healthy) people [6]. TTV infection in kidney transplant recipients has been significantly associated with markers of hepatitis B, C and E viral infections and also with the history of blood transfusion or organ transplant [11]. It is likely that there is a relationship between this virus and organ transplantation and its complications. The high prevalence of TTV in liver [12, 13] and kidney [14] transplants has been shown. Although new infections of TTV may rarely occur after transplantation [14], such prevalence [12] as well as its association with transplant complications [15] underline the importance of TTV in transplantation. 

This study was designed to further investigate the association between TTV and kidney transplantation and its late complications including post-transplant diabetes mellitus (PTDM), hyperlipidemia, hypertension, acute or chronic graft rejection, respiratory infection, urinary tract infection (UTI), cytomegalovirus (CMV) infection, BK infection, a history of delayed graft function (DGF), and the kind of immunosuppressive prescribed as maintenance (cyclosporine, tacrolimus, sirolimus). Moreover, we studied the association between TTV infection with different characteristics of the patients including the extent of HLA mismatch, glomerular filtration rate (GFR), the rate of creatinine, and having family relation with donor. Demonstrating such association may introduce the TTV infection as one of the risk factors for transplant complications.

## MATERIALS AND METHODS

**Study Subjects**

One-hundred and twenty kidney transplant recipients who had been transplanted for at least two years were enrolled in this study. The study was approved by the Ethics Committee of Kashan University of Medical Sciences. Written informed consent was obtained from all participants and their blood samples were evaluated for TTV infection. The relevance of TTV infection with later transplant complications including PCR-proven CMV infection, diabetes, hyperlipidemia, hypertension, and biopsy-proven acute and chronic rejection, were investigated. Viral, bacterial and fungal urinary and respiratory infections were confirmed by standard indicators of clinical and laboratory diagnostics for all patients.

**Blood Sample**

Three mL of blood was taken from all participants two years after transplantation. Total DNA was extracted by high pure viral nucleic acid kit (Roche, Kit Mannheim, Germany). TTV DNA was detected by two nested-PCR procedures specific for the detection of sequences included in the ORF1 region (N22 PCR) (outer primers: 1901-1918/2227-2210; inner primers: 1919-1936/2192-2175) and in the UTR region untranslated region (UTR PCR) (primers NG133/NG147; NG134/NG132) of the virus. Amplification reaction on the first round was performed with a final volume of 25 µL containing 2.5 mL DNA, 0.2 mM dNTP, 3 mM MgCl_2_, 0.2 mL of each primer sense and antisense, and 0.77 nU of Platinum™ Taq DNA polymerase. After initial DNA denaturation for 90 sec at 97 °C, amplification was done in 35 cycles of 94 °C for 30 sec, 59 °C for 30 sec, and 72 °C for 3 min and 30 sec, followed by 10 min at 72 °C, as the elongation time. The second round was performed with 1 µL of the first PCR product and 0.5 U of Platinum™ Taq DNA polymerase. The initial DNA denaturation step was done at 94 °C for 3 min, and then the sample was chased by amplification in 30 cycles of 94 °C for 30 sec, 59 °C for 30 sec, 72 °C for 75 sec, and 7 min at 72 °C, as the elongation time. Amplified PCR products obtained after the second round of PCR were run on a gel electrophoresis using 3% agarose containing ethidium bromide. UV trans-illuminator (Promega Inc, USA) was used to identify the expected 110-bp bands (with reference to the standard molecular weight) [16].

**Statistical Analysis**

The quantitative variables were expressed as mean±SD. The qualitative variables were expressed as count and percentage. Independent-sample Student’s t test was used to compare quantitative variables. Qualitative data were examined with χ^2^ test with and without Yate’s correction. Multiple binary logistic regression analysis was used to identify the independent risk factors. A p value <0.05 was considered statistically significant.

## RESULTS

We studied 120 recipients (54 women) who had undergone kidney transplantation at least two years prior to the study. The prevalence of TTV infection in kidney transplant recipients was 34.2% (95% CI: 25.6%–42.7%). Basic and laboratory findings of kidney transplant recipients with or without TTV infection are summarized in Table 1. Except for ALT, we did not find any significant differences between the two groups.

**Table 1:** Mean±SD of demographic and laboratory findings in kidney transplant recipients with and without TTV infection

Variable	TTV Negative(n=79)	TTV Positive(n=41)	p value
Age	45.5±12.0	45.2±11.0	0.339
Hemoglobin	11.6±2.1	12.5±2.1	0.739
Potassium	4.4±0.5	4.3±0.4	0.117
Platelet (×10^3^)	191.0±64.6	188.0±59.8	0.758
WBC	8178±3259	7601±2812	0.475
LDL	123.4±44.7	131.1±49.8	0.548
Systolic BP	118±14	119±15	0.945
Diastolic BP	78±9	80±10	0.523
ALT	38.5±18.1	46.2±34.5	0.018
AST	31.9±13.6	32.0±15.1	0.694

Table 2 shows the frequency of different complications, prescribed immunosuppressive drugs and other characteristics of the patients during two years post-transplantation. TTV infection had a significant association with the complications of PTDM (p=0.007), UTI (P=0.006), CMV infection (p=0.021), and graft rejection (p<0.001). It had no association with other complications such as hypertension, hyperlipidemia, and respiratory tract infection. TTV infection had a significant (p=0.027) association with prescribed drugs too—those who were using tacrolimus had lower rates of TTV infection compared to others. Serum creatinine levels more than 1.5 mg/dL (p=0.006) and DGF (p<0.001) were also associated with TTV infection.

**Table 2:** Frequency (%) of kidney transplant complications, kind of immunosuppressives prescribed and different characteristics of the patients stratified by presence or absence of TTV infection

Complication		TTV Negative (n=79)	TTV Positive (n=41)	p value
Infection	CMV infection	17 (22%)	17 (42%)	0.021
	BK infection	5 (6%)	4 (10%)	0.489
	UTI	40 (51%)	32 (78%)	0.006
	Respiratory infection	17 (22%)	14 (34%)	0.134
Drug	Cyclosporine	46 (58%)	33 (81%)	0.027
	Sirolimus	16 (20%)	6 (15%)	
	Tacrolimus	17 (22%)	2 (5%)	
DGF		7 (9%)	18 (44%)	<0.001
Mean±SD GFR		51.4±17.3	45.7±16.6	0.082
PTDM		2 (3%)	7(17.1%)	0.007
Mean±SD HLA mismatch		3.7±0.9	3.5±1.0	0.544
Rejection		3 (4%)	24 (59%)	<0.001
Cr >1.5 mg/dL		24 (30%)	23 (56%)	0.006
Positive family relation		25 (32%)	9 (22%)	0.293

Multiple binary logistic regression analysis showed that the correlation between TTV infection and cyclosporine, post-transplant rejection, positive family relation, and HLA mismatch remained significant after adjusting for confounders. Although not found inh univariate analysis, TTV infection was also found to have a significant association with family relation and HLA mismatch (Table 3).

**Table 3:** Results of logistic regression model affecting TTV infection

Variables	B	SE	p value
Cyclosporine	1.787	0.811	0.027
Sirolimus	1.679	0.929	0.071
Rejection	3.995	0.771	0.000
Positive family relation	-1.234	0.610	0.043
HLA mismatch	-0.779	0.209	0.000

## DISCUSSION

TTV infection has a worldwide distribution and is organized in different phylogenetic groups [17]. No epidemiological study has yet been done on TTV and its complications in renal transplant recipients in Iran. Given that certain segments of the viral TTV DNA are less sensitive to the PCR technique [18, 19], we used a reliable PCR technique to determine the frequency of TTV DNA in the peripheral blood of a group of Iranian renal transplant recipients. Actually, the choice of primers used in the PCR reaction, to identify the presence of TTV, can strongly affect the level of its detection in biological fluids.

We found a prevalence of 34.2% of TTV infection in kidney transplant recipients, which was similar to some studies [5, 6]. However, there are other studies demonstrating higher prevalence of TTV. For example, two Brazilian [20], and Japanese [21] studies reported a prevalence of 53.8%, and 66%, respectively. Such differences may be due to higher prevalence of TTV in their general population. Actually, there are different patterns of virus spreading in different geographic places. Another reason legitimizing such difference is different PCR methods used by investigators, which may significantly influence the results of prevalence studies. And, finally according to our results, the kind of immunosuppressive drugs used by patients may make such differences. 

In our study, no significant association was found between TTV infection and a wide variety of epidemiological and laboratory variables including age, hemoglobin level, serum potassium, platelet count, white blood cell count, serum LDL level, systolic and diastolic blood pressure, and AST activity. Similar to ours, Takemoto, *et al* [15], showed no significant association between TTV and any considered epidemiological variables including sex, blood transfusions, time of dialysis and the presence of hepatitis B in renal-transplant recipients. Importantly, we found significantly higher levels of ALT in TTV-infected recipients. Such liver enzyme abnormalities were also reported in TTV-infected patients on hemodialysis [22], emphasizing the possibility of liver dysfunction caused by TTV infection. However, in Yokosuka, *et al*, study [20], the existence of TTV had no distinctive correlation with ALT abnormality.

In line with some studies, we also found an association between post-transplantation complications and TTV infection [23-25]. Although TTV infection may be implicated in post-transplant complications, some studies have not shown such association in hemodialysis-related complications [6]. Such difference may be originated from the pathophysiologic entity of complications implicated in each condition.

As it seems foreseeable, the history of blood transfusion may be a predictor of TTV infection [6]. However, some studies have not shown such association [23, 25]. Abraham, *et al* [26], showed no correlation between TTV and its risk factors like transfusions, number of hemodialysis sessions and time duration after transplantation. It means that although the transfusion or transplantation may be a conventional route of virus transmission, there are other ways for entry of the virus. In this case, duration of dialysis and the time after transplantation may have no effect on TTV infection [23].

Interestingly, TTV infection had a considerable relevancy with important complication of DGF, graft rejection and PTDM in our study and others [23]. Considering rejection as the main cause of graft loss, we should pay more attention to such virus as an important predictor of graft outcome. Our patients using tacrolimus showed less rates of TTV infection. Therefore, in the case of drugs used, it seems that tacrolimus in better option compared to cyclosporine according to TTV infection. 

In conclusion, given that about one-third of Iranian renal transplant recipients are infected with TTV, our results should be confirmed by other multicenter studies. These people are at more risk for post-transplantation complications. In this case, we may consider TTV screening in routine laboratory studies before and after the kidney transplantation.
